# Integrated transcriptomic and metabolomic analysis identifies host response mechanisms to oncogenic Marek’s disease virus in Wenchang chickens

**DOI:** 10.1186/s13567-025-01618-5

**Published:** 2025-10-07

**Authors:** Xiangdong Xu, Junming Zhao, Sanchun He, Yuting Fang, Dan Liu, Tingbo Shen, Lifeng Zhi, Zheng Liu, Liguang Shi, Guanyu Hou, Runfeng Zhang, Guang Rong

**Affiliations:** 1 Tropical Crops Genetic Resources Institute, Chinese Academy of Tropical Agricultural Sciences, Hainan Province, Haikou, China; 2 Huangshi Biomedicine Industry and Technology Research Institute, Hubei Key Laboratory of Edible Wild Plants Conservation and Utilization, College of Life Sciences, Hubei Normal University, Huangshi, Hubei Province, China; 3 Key Lab of Tropical Crops Information Technology Application Research of Hainan Province, Institute of Science and Technology Information, Chinese Academy of Tropical Agricultural Sciences, Haikou, Hainan Province China; 4 Agriculture and Rural Affairs Bureau of Yangxin County, Huangshi, Hubei Province China

**Keywords:** Marek’s disease, host response, transcriptomics, metabolomics, chicken

## Abstract

Marek’s disease (MD), caused by Marek’s disease virus (MDV), poses a significant threat to the poultry industry worldwide by inducing neurological disorders and malignant lymphoma in infected chickens. The underlying mechanisms of the host response to MDV infection and tumorigenesis remain poorly understood. To gain insight into the host response, we analysed the transcriptomic and metabolomic profiles of the heart tissue of Wenchang chickens, an indigenous Chinese breed, using RNA sequencing and untargeted metabolomics. A total of 2470 and 2666 genes were significantly up- and down-regulated, respectively, between infected and uninfected chickens. KEGG pathway enrichment analysis revealed distinct transcriptional patterns in response to MDV infection: upregulated genes were enriched primarily in immunity-related pathways, whereas downregulated genes were associated with metabolic pathways. Among the 433 differentially expressed metabolites identified, only the caffeine metabolism pathway approached statistical significance (*p* = 0.067). Integrative mapping of genes and metabolites to the KEGG enzyme database highlighted L-tryptophan interactions, with KYNU, KMO, KYAT3, and AADAT as the most representative relationships. These results provide a quantitative overview of MDV-induced transcriptional and metabolic perturbations, suggesting that hosts may counteract viral infection and tumor progression by suppressing cellular metabolism to potentiate immune responses.

**Supplementary Information:**

The online version contains supplementary material available at 10.1186/s13567-025-01618-5.

## Introduction

Marek’s disease (MD) is a major health problem in poultry production systems worldwide and causes an annual global economic loss of approximately $2 billion [1]. MD is a complex immunosuppressive and lymphoproliferative disease of chickens. The pathological features of MD include paralysis, neuroinflammation, and chronic depletion, as well as visceral and muscle lymphomas in chickens [2–4]. The causal agent is a naturally occurring α-herpesvirus with double-stranded DNA called Gallid alphaherpesvirus 2 (GaHV2), commonly referred to as Marek's disease herpesvirus (MDV). MDV toxicity progresses from mildly virulent (m), virulent (v), and very virulent (vv) to very virulent plus (vv +) [5]. Invasion by virulent and very virulent strains causes transient paralysis in most breeds, and very virulent plus strains cause brain damage and eventually death [6]. Moreover, as the virus evolves, the virulence of MDV gradually increases [5]. In the “Cornell model” [7], MDV undergoes a long and complex pathogenic life cycle involving four phases: (a) the early cytolytic phase (2–7 days post-infection (dpi)), (b) the latent phase (from 7 to 10 dpi), (c) the late cytolytic and immunosuppressive phase (from 18 dpi) and (d) the proliferative phase (from 28 dpi).

MDV infection induces a complex process that activates various components of the host immune system along with virus-induced physiological changes and ultimately leads to lymphoid tumours in susceptible chickens. Considerable efforts have been made in recent years to investigate the basis of host immune responses to MDV infection, with a focus on the experimental White Leghorn (layer) lines 6_3_ and 7_2_, which are MD resistant and susceptible, respectively. Genome-wide QTL mapping studies have identified loci or domains associated with MD resistance or susceptibility on chromosomes 1, 5, 7, 9, 15, 18, 26, Z, E21, and E16 [8–14]. Available chicken microarrays have become useful tools to study the gene and protein expression profiles of MDV-host interactions and to further elucidate the molecular mechanisms involved in pathogenic and tumorigenic responses to MDV infection. Genome-wide microarray analyses revealed genes with dysregulated expression in response to MDV exposure in embryonic fibroblasts, peripheral blood leukocytes, lymph nodes, or chicken epithelial tissues [13, 15–21]. These candidate genes are involved in many biological processes involved in host‒pathogen interactions, such as antigen presentation, the interferon (IFN) response, signal transduction, the cytoskeleton, the immune response, cell surface molecules, and apoptosis. Recently, several studies have employed RNA sequencing technology coupled with quantitative real-time PCR to explore the dynamic and differential gene expression profiles of the liver, thymus, spleen, or bursa of Fabricius of MDV-infected chickens [22–25] and revealed more than 5000 genes with dysregulated expression at different stages upon induction of MDV exposure.

MDV uses and reshapes the metabolic network of the host to facilitate its own replication and assembly. MDV infection can increase arginase activity in macrophages, altering the metabolism of various amino acids, including arginine, which play a key role in promoting tumorigenesis [26]. Moreover, MDV infection can regulate the metabolic reprogramming of host cell glycolysis by increasing the activity of mitochondrial fatty acid β-oxidation, which can increase the oxygen consumption rate in MDV-infected cells, ultimately leading to an increase in glycolysis to promote virus replication [27]. MDV infection can significantly alter metabolites in chicken embryo fibroblasts, with most changes occurring in amino acid metabolism, energy metabolism, nucleotide metabolism, and lipid metabolism, especially the upregulation of amino acids in host cells during the early stages of infection to provide the energy and intermediary metabolites necessary for efficient multiplication of its own replication [28]. The modification of metabolic signalling pathways in host cells during the infection process is evident, which may have a significant impact on the pathogenesis of MDV.

Although these studies have expanded our understanding of MDV infection, the molecular mechanisms of host–virus interactions in MD are far from fully understood. Further studies are necessary to elucidate the exact mechanisms by which the host responds to virus infection in terms of immunity and metabolism, thereby providing deeper insight into this fascinating biological phenomenon. In this study, we employed RNA sequencing (RNA-seq) and an untargeted metabolic approach to discern transcriptomic and metabolomic changes in chickens naturally infected with MDV. Our findings demonstrated that many genes and metabolites are significantly altered during MDV infection. These alterations suggest potential molecular mechanisms of the host immune response along with MD pathogenesis and tumorigenesis.

## Materials and methods

### Animals and sample collection

The broiler Wenchang birds hatched in the same batch were kept and managed in two separate houses under the same conditions at the farming base of Yexiang Wenchang Chicken Breeding Co., Ltd., in Hainan Province. At 60 days of age, 30% of the birds presented classic clinical signs of Marek’s disease, including lethargy, glazed eyes, paralysis, anorexia, and pathological emaciation. Symptomatic chickens were immediately transferred to isolated houses, and asymptomatic chickens remained in the original facility. At 70 days of age, five chickens that exhibited clinical symptoms and five asymptomatic chickens were humanely euthanized via CO₂ asphyxiation (30% chamber volume/min) followed by confirmatory cervical dislocation. Three subsamples from the same heart tissue were taken for MDV detection, RNA-seq and metabolomic analysis and quickly frozen in liquid nitrogen for further use. At the same time, anticoagulant blood samples were collected for detection of avian leukosis virus (ALV). The animal experiments were approved by the Animal Care and Use Committee of the Chinese Academy of Tropical Agricultural Sciences and were conducted in accordance with the Guidelines for Experimental Animals of the Ministry of Science and Technology (Beijing, China).

### Viral infection detection

ELISA and PCR were performed to detect ALV and/or MDV infection in these two groups according to the Chinese ALV disease diagnostic methods and the Chinese Marek’s disease diagnostic methods (GT/B 26436-2010 and GBT18643-2021), respectively.

### Transcriptomic analysis and quantitative real-time polymerase chain reaction (qRT‒PCR)

Total RNA from frozen heart tissues was extracted using TRIzol reagent (Invitrogen, Carlsbad, CA, USA) following the manufacturer’s instructions. The RNA content of each sample was then determined using a NanoDrop One (Thermo Fisher Scientific, MA, USA). The quality of the RNAs was then determined using an Agilent 2100 Bioanalyzer (Agilent Technologies, Santa Clara, CA, USA), and RNA integrity was measured by denatured agarose gel electrophoresis. The extracted RNA was then prepared using the NEBNext Ultra Directional RNA Library Prep Kit (New England Biolabs, Ipswich, MA, USA). Libraries were subjected to 150 bp paired-end sequencing on an Illumina HiSeq2000 sequencer.

Clean reads were obtained from raw sequencing reads after removing sequencing adaptors, duplicated sequences, and low-quality reads and then mapped against the chicken reference genome Gallus_gallus. GRCg6a (version 105.6) was generated via HISAT2 (v2.0.5) with the default parameters [29]. The uniquely mapped reads were counted to calculate the number of reads per gene using HTSeq (2.0.2) [30]. Differentially expressed genes (DEGs) were identified between the infected and uninfected groups using DESeq2 [31] and then filtered with a false discovery rate (FDR) ≤ 0.05 and |log_2_FoldChange |≥ 1. To better understand the functional involvement of these DEGs, topGO [32] and clusterProfiler [33] were used for GO and KEGG pathway enrichment analysis. The DEG network was developed using NetworkAnalyst [34], which is based on protein‒protein interactions derived from SRTING (Interacting Genes/Proteins, version 11.5).

Ten genes were chosen to validate the results obtained from the RNA-seq analysis by qRT-PCR (Additional file 1). Complementary DNA (cDNA) was synthesized from total RNA using HiScript^®^ Q RT SuperMix for qPCR (+ gDNA wiper) (Vazyme, Nanjing, China) according to the manufacturer’s instructions. All PCR reactions were carried out in a QuantStudio™ 5 Real-Time PCR System (Applied Biosystems, USA). The reactions were run in triplicate in a total volume of 10 μL containing the following: 2.0 μL of cDNA (100 ng), 0.25 μL of each primer (forward and reverse, 10 nM each), 5.0 μL of 2 × ChamQ SYBR qPCR Master Mix (Vazyme, Nanjing, China) and 2.5 μL of nuclease-free water. The amplification reactions were subjected to initial denaturation at 95 ℃ for 30 s, followed by 40 cycles of denaturation at 95 ℃ for 10 s, annealing at 60 ℃ for 30 s, and 72 ℃ for 20 s. The relative gene expression of each target gene was calculated using the 2^−ΔΔCt^ method with glyceraldehyde-3-phosphate dehydrogenase (GAPDH) as the internal reference.

### Metabolomic analysis

Flesh samples were thawed at 4 ℃ and homogenized using a grinding pestle. A 100 μL aliquot of the homogenized sample was transferred into a 2 mL centrifuge tube containing 1000 μL of methanol, followed by grinding for 1 min. The sample was then centrifuged at 12,000 rpm for 10 min at 4 °C. The supernatant was transferred to a new 2 mL centrifuge tube and concentrated until drying. The residue was reconstituted in 200 μL of 2-chloro-L-phenylalanine (4 ppm) solution prepared in 80% methanol water (stored at 4 ℃), filtered through a 0.22 μm membrane, and transferred to a detection bottle for liquid chromatography‒tandem mass spectrometry (LC‒MS) analysis.

Metabolite analysis of the heart tissues was conducted by PANOMIX (Suzhou, China) using an LC‒MS system comprising a Vanquish UHPLC System and Orbitrap Exploris 120 mass spectrometer (Thermo Fisher Scientific, USA). The extracts were injected onto an ACQUITY UPLC^®^ HSS T3 column (2.1 × 100 mm, 1.8 μm, Waters, Milford, MA, USA), which was maintained at 40 °C. The temperature, flow rate and injection volume of the automatic injector were set at 4 °C, 0.3 mL/min and 2 μL, respectively [35]. Mass spectrometric detection of metabolites was performed on an Orbitrap Exploris 120 (Thermo Fisher Scientific, USA) with an ESI ion source [36]. Simultaneous MS1 and MS/MS (full MS‒ddMS2 mode, data-dependent MS/MS) acquisition was used. Metabolite identification was performed with high precision, initiated by accurate m/z measurements followed by MS/MS fragmentation pattern analysis. Cross-referencing of the identified metabolites was performed against the Human Metabolome Database (HMDB), MassBank, LipidMaps, mzCloud, and the metabolite database built by PANOMIX (Shuzhou, China) to confirm and validate the findings. Two different multivariate statistical analysis models, unsupervised and supervised, were applied to discriminate the groups (PCA; PLS-DA; OPLS-DA) by Ropls (v1.22.0) package [37]. Differentially expressed metabolites (DEMs) were selected (VIP > 1 and *p* value < 0.05). KEGG and MetaboAnalyst [38] were used to search for metabolite pathways.

### Transcriptome and metabolome association analysis

The associations between DEGs and DEMs were analysed on the basis of the Pearson correlation coefficient. All DEGs and metabolites were mapped to the KEGG pathway database, and their common pathway information was obtained. The main biochemical and signal transduction pathways were subsequently analysed. The networks of genes and metabolites were analysed by igraph software [39].

## Results

### Viral infection identification

ELISA of the ALV p27 antigen revealed that the OD values of all the samples were less than 0.2 (the OD value for the negative control was less than 0.2, and that for the positive control was greater than 0.8) after the DF-1 cells were infected with the viral mixture and cultured for 7 days. These results indicate that these individuals are negative for ALV infection.

The PCR results revealed that a specific fragment of 786 bp for the MDV *meq* gene and a specific fragment of 317 bp for MDV 132 *bpr* were amplified from samples of sick birds, whereas neither of the two fragments were amplified from samples of healthy birds, confirming that those sick birds were infected by MDV (Additional file 2).

### Transcriptomic analysis

**Figure 1:**
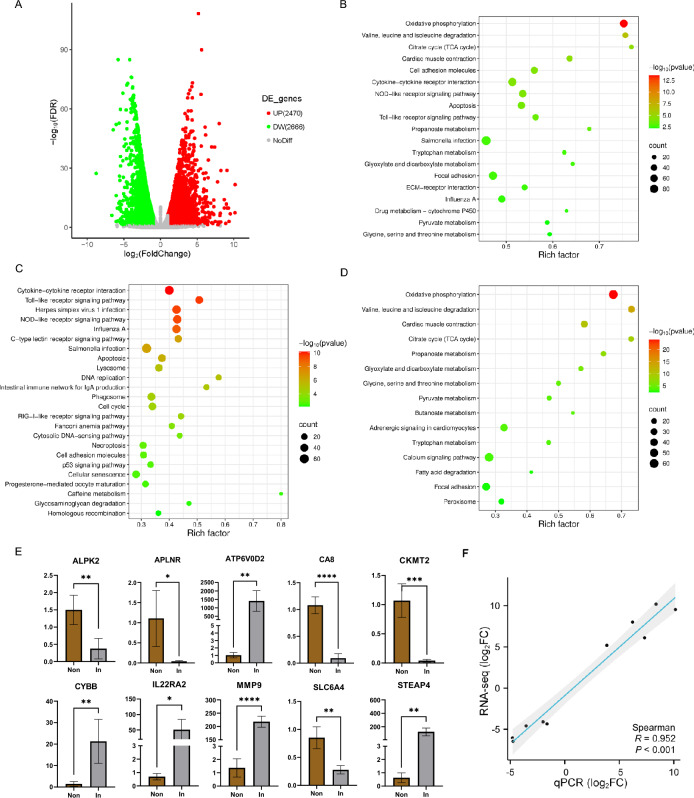
**Transcriptomic pattern variation in the heart tissues of Wenchang chickens infected with MDV**. **A** Volcano plot. The red and green colours indicate genes detected as up- and down-regulated in the infected group compared with the uninfected group at FDR < 0.05. **B** Bubble plots for KEGG pathways enrichment of DEGs. **C** Bubble plots for KEGG pathways enrichment of upregulated DEGs. **D** Bubble plots for KEGG pathways enrichment of downregulated DEGs. **E** Real-Time RT-PCR analysis of selected genes. Relative expression levels calculated from standard curves were normalized to the endogenous control GAPDH gene. Numbers represent mean plus standard error. ALPK2: alpha kinase 2; APLNR: apelin receptor; ATP6V0D2: ATPase H + transporting V0 subunit d2; CA8: carbonic anhydrase 8; CKMT2: creatine kinase, mitochondrial 2; CYBB: cytochrome b-245 beta chain; IL22RA2: interleukin 22 receptor subunit alpha 2; MMP9: matrix metalloproteinase 9; SLC6A4: solute carrier family 6 member 4; STEAP4: Six-transmembrane epithelial antigen of the prostate 4. *p < 0.05; **p < 0.01. **F** Correlation of fold changes calculated from qPCR and RNAseq analysis. Black dots represent log_2_ transformed fold change values of a single gene in an infected sample obtained from qPCR (X-axis) and RNAseq analysis (Y-axis). R: correlation coefficient.

Ten cDNA libraries were constructed for the hearts of infected and uninfected chickens, resulting in 60.39 GB of clean reads, including 400 004 580 reads after quality control assessment. Approximately 97.68% of the raw reads (± 2.96%, SD) were uniquely mapped to the chicken genome assembly. A total of 5,136 DEGs were identified between the infected and uninfected groups (FDR ≤ 0.05 and |log_2_FoldChange |≥ 1). Compared with the uninfected group, the infected group presented 2470 and 2666 up- and down-regulated genes, respectively (Figure 1A, Additional file 3). All DEGs were mapped to KEGG pathways, and 19 significantly enriched pathways were obtained (Figure 1B, Additional file 4), among which Salmonella infection, cytokine‒cytokine receptor interaction, focal adhesion, oxidative phosphorylation, and the NOD-like receptor signalling pathway were the five most representative pathways. The upregulated DEGs were significantly enriched in 24 pathways related mainly to the immune system, cell growth and death, and infectious diseases, including the Toll-like receptor signalling pathway, NOD-like receptor signalling pathway, apoptosis, lysosome, herpes simplex virus 1 infection, and influenza A (Figure 1B). The downregulated DEGs were mostly related to carbohydrate metabolism, the circulatory system and amino acid metabolism, as shown by enriched KEGG pathways such as oxidative phosphorylation; valine, leucine and isoleucine degradation; myocardial contraction; the citrate cycle (TCA cycle); and propanoate metabolism (Figure 1B).

The RNA-seq results of selected genes were validated in the same sample set by qRT‒PCR (Figure 1C). The correlation coefficient (R) of the log_2_-transformed fold change values between the qRT‒PCR and RNA‒seq platforms was 0.952 (Figure 1D). The expression results of ALPK2, APLNR, ATP6V0D2, CA8, CKMT2, CYBB, IL22RA2, MMP9, SLC6A4, and STEAP4 obtained by qRT‒PCR were in good agreement with the RNA‒seq results (*p* < 0.001).

### Metabolomic analysis

**Figure 2:**
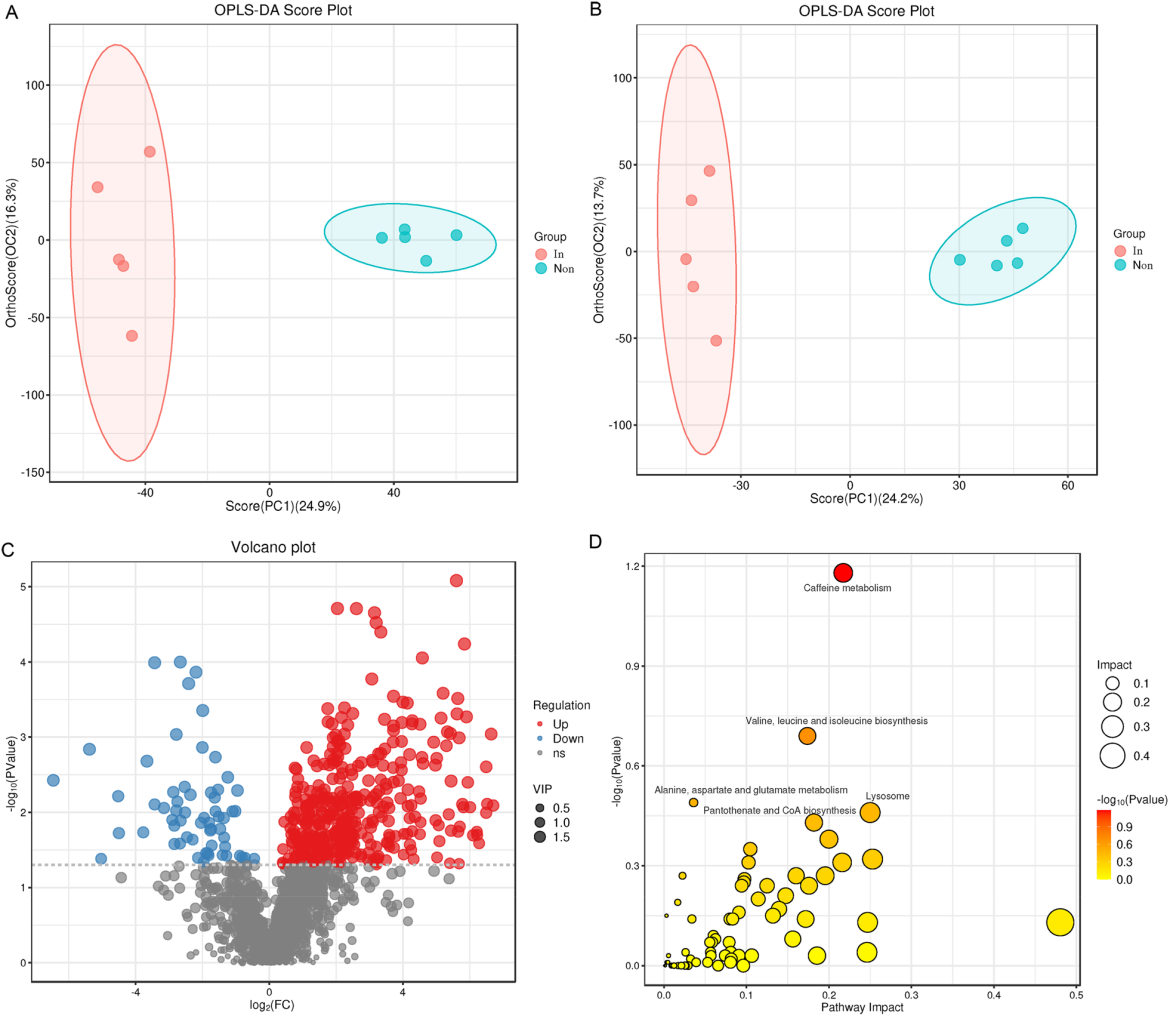
**Metabolomic pattern variation in the heart tissues of Wenchang chickens infected with MDV.** **A** OPLS-DA score plot of positive ions. **B** OPLS-DA score plot of negative ions. **C** Volcano plot of DEMs. **D** KEGG pathway enrichment of DEMs.

Multivariate statistical analysis revealed a clear separation of metabolites between the infected and noninfected groups in the negative model (Figure 2A) and the cation model (Figure 2B). A total of 433 DEMs (261 negative model and 172 positive model) were identified by setting thresholds of VIP > 1 and *p* value < 0.05, including 372 upregulated and 61 downregulated DEMs (Figure 2C, Additional file 5). Among these DEMs, dihydroxyfumitremorgin C (log_2_FoldChange = 6.7) and L-kynurenine (log_2_FoldChange = 6.64) presented the greatest increases in the positive and negative models, respectively, whereas pantetheine 4'-phosphate (log_2_FoldChange = −4.52) and 2-biphenylol (log_2_FoldChange = −6.46) presented the lowest decreases in infected birds. All the DEMs were subjected to KEGG annotation analysis, and only the caffeine metabolism pathway was nearly significantly enriched (*p* = 0.067) (Figure 2D, Additional file 6).

### Comprehensive analysis of transcriptomics and metabolomics

**Figure 3:**
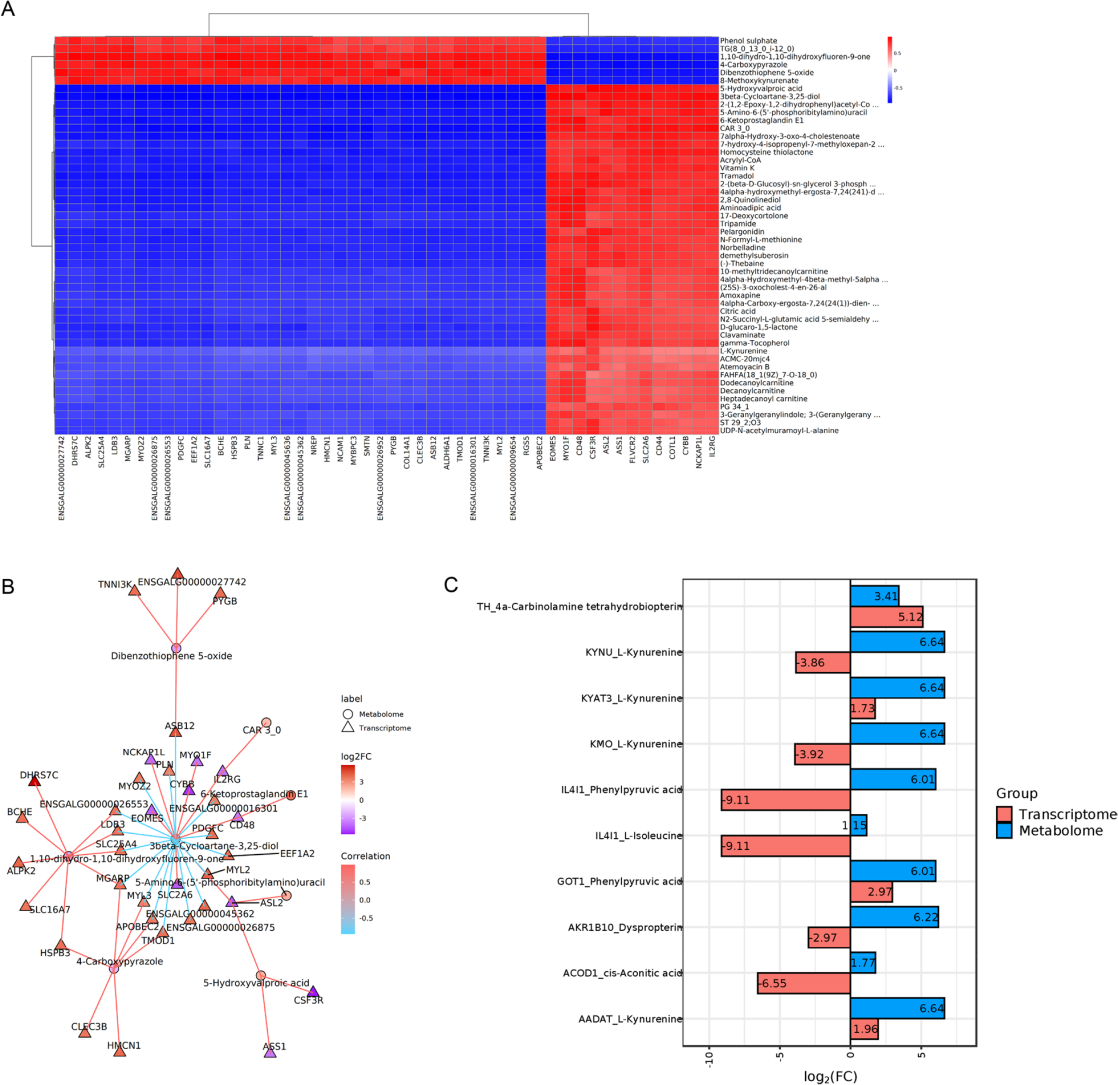
**Comprehensive Analysis of Transcriptomics and Metabolomics.** **A** Correlation heatmap of the top 50 significantly expressed genes and significantly expressed metabolites. **B** Correlation network of the top 50 significantly expressed genes and significantly expressed metabolites. **C** Top 10 corresponding relationships between metabolites in metabolic processes and the expression regulation of enzymes.

Through Pearson correlation analysis, correlations between differentially expressed genes (DEGs) from transcriptomics and differentially expressed metabolites (DEMs) from metabolomics were calculated. Correlation coefficients greater than 0 indicated positive relationships, whereas values less than 0 represented negative associations. Gene‒metabolite pairs with correlation coefficients > 0.8 and *p* values < 0.05 were classified as strongly correlated. The correlation heatmaps and interaction networks of the top 50 differentially abundant metabolites and genes are presented in Figures 3A and B (Additional file 7). These results demonstrated significant transcript‒metabolite interactions. Both DEGs and DEMs were annotated against the KEGG pathway database, but no shared significantly enriched pathways were identified. To further investigate enzyme‒metabolite regulatory relationships, DEGs and DEMs were mapped to the KEGG enzyme database. The top 10 associations ranked by |log_2_FoldChange| magnitude are displayed in Figure 3C (Additional file 8). Among these, L-kynurenine paired with KYNU, KMO, KYAT3, and AADAT emerged as the most representative interaction.

## Discussion

Previous studies have documented the global gene expression response to MDV infection in chickens [13, 15, 17, 18, 20–23, 40, 41]. Although these analyses provide valuable and important insights into host responses and virus‒host interactions, their scope is limited by the coverage of the gene arrays used and the use of highly selected breeds/lines that show differential resistance to MDV challenges under laboratory conditions. In this study, we further expanded the current knowledge of host transcriptomic and metabolomic responses to MDV infection by comparing the transcriptome and metabolome changes in the Chinese native breed Wenchang chicken under natural conditions via RNA-seq and untargeted metabolomics technology. This study focused mainly on the tumor transformation stage after MDV infection. Our results revealed distinct transcriptomic and metabolomic patterns that may confer an immune response to MD.

Close examination of the transcriptomic changes in response to MDV infection revealed that immune-related genes (IRGs) were activated in infected Wenchang chickens. Compared with the list of 1546 IRGs downloaded from the Gene Ontology Resource, the KEGG pathway database and DisGeNET, a total of 651 IRGs were significantly impacted by MDV infection in this study, including 459 upregulated and 192 downregulated genes (Additional file 9). Among these genes, many have been previously described in studies of the host response to MDV exposure [13, 15, 17, 18, 20–23, 40, 41], including genes related to chemokines (CCL19 and CXCL13L2), cytokines (IL21R, IL2RA), the innate immune response (IRF1, STAT1, CD36, IFIH1, SLC11A1, GCH1), and the adaptive immune response (B2M, P2RX7, SLC11A1). This overlap confirms that these candidate genes may play important roles in the response to MDV challenge. On the other hand, the six genes differentially expressed in this study (F13A1, HSP90AB1, RGS5, MMP7, IL4I1, and ADAMTS2) were promising top candidate genes conferring genetic resistance to MD [41]. In addition, certain genes impacted in this study have been implicated in MDV resistance/susceptibility in previous studies. These include CTLA4, CD72, TRANK1, SOCS1, TLR4, CD7, EDN1, IL1B, IFNG, CD79B, and PTPN3 [12, 41, 42].

A list of 1325 genes has been mapped to quantitative trait loci regions for chickens resistant to MDV [10, 12–14, 40, ChickenQTLdb]. A total of 263 DEGs in this study, accounting for 19.85% of the 1325 quantitative trait region genes, were identified on the basis of their locations within QTL regions and their expression patterns after MDV infection, including EOMES, FST, CSTA, C1S, CSTA, C1S, LAG3, CTNNA2, CD79B, TRANK1, SOCS1, TLR4, and CD7 [12, 41]. Notably, six members of the B7 family and cell adhesion molecules (CD274, PDCD1LG2, CD86, CD80, DMA and CD4) were significantly induced by infection, whereas eight genes involved in oxidative phosphorylation and the mitochondrial respiratory chain (UQCRFS1, UQCRC2, UQCRC1, ATP5G3, ATP5PF, NDUFB6, SUCLG1 and PRDX3) were significantly reduced in infected birds. The eight pathways significantly enriched by these 263 genes were related mainly to immunity, and cell adhesion molecules; glycine, serine and threonine metabolism; and the intestinal immune network for IgA production were the most representative pathways (Additional file 10). The 112 upregulated genes were significantly mapped into three pathways of the intestinal immune network for IgA production, cell adhesion molecules, and the Toll-like receptor signalling pathway, whereas the 151 downregulated genes were markedly enriched in eight pathways involved in metabolism, including glycine, serine and threonine metabolism; propanoate metabolism; glyoxylate and dicarboxylate metabolism; tryptophan metabolism; and pyruvate metabolism.

Many studies have revealed how MDV cleverly uses and reshapes the metabolic network of host cells to facilitate its own replication and assembly [27, 28, 43, 44]. In infected cells, viral replication relies heavily on the host machinery to produce large quantities of viral macromolecules and virus particles. The synthesis of the building blocks of viral particle-nucleotides, viral proteins, amino acids, lipid acids and genomes and the transport of viral components in the cell require energy, typically in the form of ATP. In addition to the downregulation of fatty acid metabolism, many downregulated genes were enriched in cellular respiration-related biological processes, such as cellular respiration, the respiratory electron transport chain, ATP synthesis coupled with electron transport, and mitochondrial respiratory chain complex assembly, in this study. Thus, many metabolic pathways, including valine, leucine and isoleucine degradation; glycine, serine and threonine metabolism; fatty acid degradation; the citrate cycle (TCA cycle); and pyruvate metabolism, were significantly enriched among the downregulated DEGs. Glycolysis is a major metabolic pathway in the cytoplasm that produces not only ATP but also metabolites for biosynthetic pathways such as the synthesis of lipids, amino acids, and nucleic acids. Pyruvate occupies a central metabolic node by virtue of its position at the crossroads of glycolysis and the tricarboxylic acid (TCA) cycle through the malic enzyme pathway to generate NADPH to support lipid biosynthesis. Pyruvate kinase catalyzes the final step of glycolysis, the production of pyruvate and ATP from glucose. In infected cells, glycolytic pyruvate kinase is directly recruited to the viral replicase complex to generate ATP for RNA synthesis [45]. Thus, we propose that the host may actively respond to MDV infection and tumor progression by regulating the balance between immunity and metabolism, which needs further investigation.

Mapping of differentially expressed genes and metabolites to the KEGG database revealed that L-kynurenine pairing with KYNU, KMO, KYAT3 and AADAT may function in host–virus interactions during MDV infection and tumor progression in cardiac tissue. Tryptophan is metabolized primarily through the kynurenine pathway, where it is converted to N-formyl-kynurenine by indoleamine-2,3-dioxygenase (IDO1 or IDO2) and tryptophan-2,3-dioxygenase (TDO2) and then demethylated to form kynurenine. Kynurenine is processed into either kynurenic acid or NAD + through two main branches of the kynurenine pathway. In the NAD + branch pathway, kynurenine is converted to NAD + via three different enzymes: kynurenine 3-monooxygenase (KMO), kynurenineinase (KYNU), and 3-hydroxyanthranilic acid dioxygenase (HAAO) [46]. Recent studies have shown that kynurenine, a potent immunomodulatory molecule, inhibits the proliferation and activity of T cells and natural killer cells and promotes the differentiation of regulatory T cells [47–50]. Hyperactivation of the kynurenine pathway promotes cancer cell invasion, metastasis, and chemoresistance and is associated with poor prognosis in a variety of cancers [51–54]. KMO, which uses NADPH or NADH as a coenzyme, hydroxylates kynurenine to form 3-hydroxykynurenine (3HK) [55]. Studies have indicated that KMO and its product quinolinic acid have broad-spectrum antiviral effects [56], and KMO is overexpressed in the tissues of patients with triple-negative breast cancer [57], colorectal cancer [58], and liver cancer [59]. Elevated KMO expression alters the formation of 3-hydroxykynurenine and quinolinic acid and regulates immune responses and tumor tolerance, and high KMO expression is correlated with poor tumor prognosis [57–59]. KYNU, a key enzyme in the kynurenine pathway, catalyzes the formation of the metabolites anthranilic acid or 3-hydroxyanthranilic acid from kynurenine or 3-hydroxykynurenine. 3-Hydroxyphthalaminobenzoic acid is a modulator of the immune response and enhances apoptosis via iron or manganese ions in monocytes and macrophages under inflammatory conditions, triggering the death of activated T cells by depleting intracellular glutathione [60]. Moreover, 3-hydroxyanthranilic acid can inhibit dendritic cell maturation and T-cell activation [61]. Several studies have shown that KYNU expression is significantly associated with small cell lung cancer [62], triple-negative breast cancer subtypes [63], and cutaneous squamous cell carcinoma [64]. In this study, L-kynurenine abundance was significantly greater in the heart tissues of MDV-infected Wenchang chickens than in those of normal Wenchang chickens, whereas the expression of genes encoding two key enzymes related to kynurenine metabolism, KYNU and KMO, was significantly lower. It is speculated that the host downregulates the expression of the KYNU and KMO genes to enhance its immune response to MDV infection and its induced tumors. The specific mechanisms of action require further investigation.

## Supplementary Information

**Additional file 1. Sequences of Primers used for qPCR validation**.

**Additional file 2. Identification of Marek’s disease virus infection**.

**Additional file 3. DEGs**.

**Additional file 4. KEGG analysis of DEGs**.

**Additional file 5. KEGG analysis of DEGs**.

**Additional file 6. KEGG pathways associated with DEMs**.

**Additional file 7. Top 50 DEMs and DEG correlations**.

**Additional file 8. Top 10 corresponding relationships of DEMs and DEGs**.

**Additional file 9. IRGs**.

**Additional file 10. DEGs within the QTLR for MD resistance**.

## Data Availability

The raw RNA-seq data are available in the National Genomics Data Center (NGDC) Genome Sequence Archive (GSA: CRA013443). The raw metabolomic datasets in the current study are available from the corresponding author upon reasonable request.
