# Supplementary material for: Integrated transcriptomic and metabolomic analysis identifies host response mechanisms to oncogenic Marek’s disease virus in Wenchang chickens
Source: Vet Res. 2025 Oct 7;56:190. doi: 10.1186/s13567-025-01618-5 (PMC12505730; doi:10.1186/s13567-025-01618-5)
Supplement: Supplementary file 3 — Additional file 3. DEGs. [file 13567_2025_1618_MOESM3_ESM.docx]

**Additional file 2 Identification of Marek’s disease virus infection**

**Methods**

***DNA extraction and conventional PCR***

Heart samples were aseptically collected and stored at -80 °C. The tissue samples were processed according to the manufacturer’s protocol using a Blood and tissue kit (Anheal Laboratories, China). Total DNA concentration was quantified using a NanoDrop One spectrophotometer (NanoDrop Technologies, USA). Specific oligonucleotide primers for GaHV-2 *meq* and *132 bpr* gene were used to detect MDV serotypes (Table 1).

Table 1 Set of primers for conventional PCR

| Gene | Primer sequence 5'–3' | Product length | Temperature |
| --- | --- | --- | --- |
| MDV-*meq* | F: 5’-TTCCCTGACGGCCTATCTGA-3’  R: 5’-TTCGGGATCCTCGGTAAGAC-3’ | 786 /963 bp | 53 ℃ |
| MDV-*132 bpr* | F: 5’-TGCGATGAAAGTGCTATGGAGG-3’  R: 5’-GAGAATCCCTATGAGAAAGCGC-3’ | 317/449 bp | 53 ℃ |

Note: Specific oligonucleotide primers and PCR conditions in this study were processed according to GB/T 18643-2021 (Diagnostic techniques for Marek’s disease of China).

PCR conditions were optimized in a 20 μL reaction volume including 0.5 μL of Hot Start DNA polymerase (Sangon Biotech, China), 1.0 μL of forward and reverse primer, 10 μL 2 × Direct PCR Mix and 10 ng of DNA. Amplification of the MDV-*meq* and *132 bpr* was performed using 35 cycles of 98 °C for 5 s, 53 °C for 20 s min, and 72 °C for 1 min. PCR products were separated on a 1% agarose gel and stained with ethidium bromide (1 mg/mL). The bands were visualized in a UV trans-illuminator and analyzed using Bio-Rad Gel Doc™ XR+ system (BioRad, USA).

***Sequencing and phylogenetic analysis***

The *meq* gene PCR products were purified from the gel using the QIAquick gel extraction kit (Qiagen, USA) following the manufacturer’s recommendations. The purified products were sequenced by Sangon Biotech (Shanghai) Co., Ltd. Sequence data were assembled and edited using the SeqMan program (DNA Star Laser gene software package, USA). Nucleotide alignments of *meq* gene sequences were performed with MEGA X using the Muscle algorithm. A phylogenetic tree was generated using the neighbor-joining (N-J) method, with 1000 replicates. The MDV reference sequences were retrieved from the GenBank database (Table 2).

Table 2 GaHV-2 strains used for the phylogenetic analysis.

| Isolate | Accession No. | Origin | Pathotype |
| --- | --- | --- | --- |
| 686 | AY362727 | USA | vv+MDV |
| 660-A | AY362726 | USA | vv+MDV |
| 648A | AY362725 | USA | vv+MDV |
| X | AY362724 | USA | vv+MDV |
| U | AY362722 | USA | vv+MDV |
| TK | AY362721 | USA | vv+MDV |
| RL | AY362720 | USA | vv+MDV |
| New | AY362719 | USA | vv+MDV |
| N | AY362718 | USA | vv+MDV |
| L | AY362717 | USA | vv+MDV |
| 584a | DQ534532 | USA | vv+MDV |
| W | AY362723 | USA | vv+MDV |
| ATE2539 | MF431493 | Hungary | vv+MDV |
| Polen5 | MF431496 | Poland | vv+MDV |
| UDEACO04/2013 | KU058701 | Colombia | vv+MDV |
| UDEACO06/2013 | KU058696 | Colombia | vv+MDV |
| UDEACO07/2013 | KU058697 | Colombia | vv+MDV |
| 643P | AY362716 | USA | vvMDV |
| 595 | AY362715 | USA | vvMDV |
| 549 | AY362714 | USA | vvMDV |
| Js201801 | MK046676 | China | vvMDV |
| Md5 | NC_002229 | USA | vvMDV |
| RB1B | AY243332 | USA | vvMDV |
| 02LAR | EF523772 | Australia | vvMDV |
| FT158 | EF523771 | Australia | vvMDV |
| Woodlands1 | EF523775 | Australia | vvMDV |
| 567 | AY362709 | USA | vMDV |
| 571 | AY362710 | USA | vMDV |
| 573 | AY362711 | USA | vMDV |
| 617A | AY362712 | USA | vMDV |
| 637 | AY362713 | USA | vMDV |
| RB1B | DQ534541 | NA | vMDV |
| MD70/13 | MF431495 | Hungary | vMDV |
| 04CRE | EF523773 | Australia | vMDV |
| MPF57 | EF523774 | Australia | vMDV |
| BC-1 | AY362707 | USA | vMDV |
| JM | AY243331 | USA | vMDV |
| JM/102W | DQ534539 | USA | vMDV |
| CU-2 | AY362708 | USA | vMDV |
| CVI988 | DQ530348 | Netherlands | vMDV |
| 3004 | EU032468 | Russia | vMDV |
| RM-1 | DQ534542 | NA | vMDV |
| CVI988 | DQ534538 | NA | vMDV |
| 2014021 | KU382455 | China | NA |
| 2014055 | KU382456 | China | NA |
| 2013032 | KU382454 | China | NA |
| J-1 | KU744555 | China | NA |
| GX060167 | EU697887 | China | NA |
| HNGS101 | HF546084 | China | NA |
| HNGS201 | HF546085 | China | NA |

Note: 50 strains, including virulent (v), very virulent (vv), very virulent plus (vv+) pathotypes, were selected for comparison of their *meq* gene sequences. NA, not available.

**Results**

The *meq* PCR primers were used to determine the *meq* gene of MDV-1. Strains and differences in its sequence are associated with oncogenicity. As expected, an approximately 786 bp DNA fragment was amplified and visualized on an agarose gel, confirming the presence of wildtype of MDV-1 according to GB/T 18643-2021 (Diagnostic techniques for Marek’s disease of China) (Figure 1). The BamH1-H-BamH1-D-132 bp PCR amplicons analyzed by agarose gel electrophoresis reconfirmed the presence of wildtype of MDV-1 in the isolates (Figure 2). For every isolate, a band was produced corresponding to a 317 bp DNA fragment associated with wildtype MDV-1. The presence of a single band also indicated the presence of virulent MDV-1 strain(s) and the absence of the CVI988/Rispens vaccine strain according to GB/T 18643-2021 (Diagnostic techniques for Marek’s disease of China).

Partial sequencing of the *meq* gene of the isolate revealed > 99% nucleotide sequence identity to virulent and very virulent plus strains from a few countries, placing it in the same clade as a very virulent Chinese strain (Js201801, MK046676) and a very virulent plus Hungary strain (ATE2539, MF431493) (Figure 3).


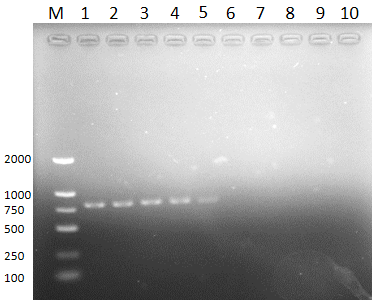


Figure 1. PCR amplification of *meq* gene of MDV-1. Lanes M: DL2000 marker, lanes 1-5: the MDV-1 infected samples, lanes 6-10: the samples from the control group.


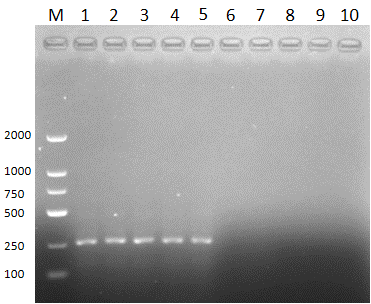


Figure 2. PCR amplification of BamH1-H-BamH1-D-132 bp of MDV-1. Lanes M: DL2000 marker, lanes 1-5: the MDV-1 infected samples, lanes 6-10: the samples from the control group.


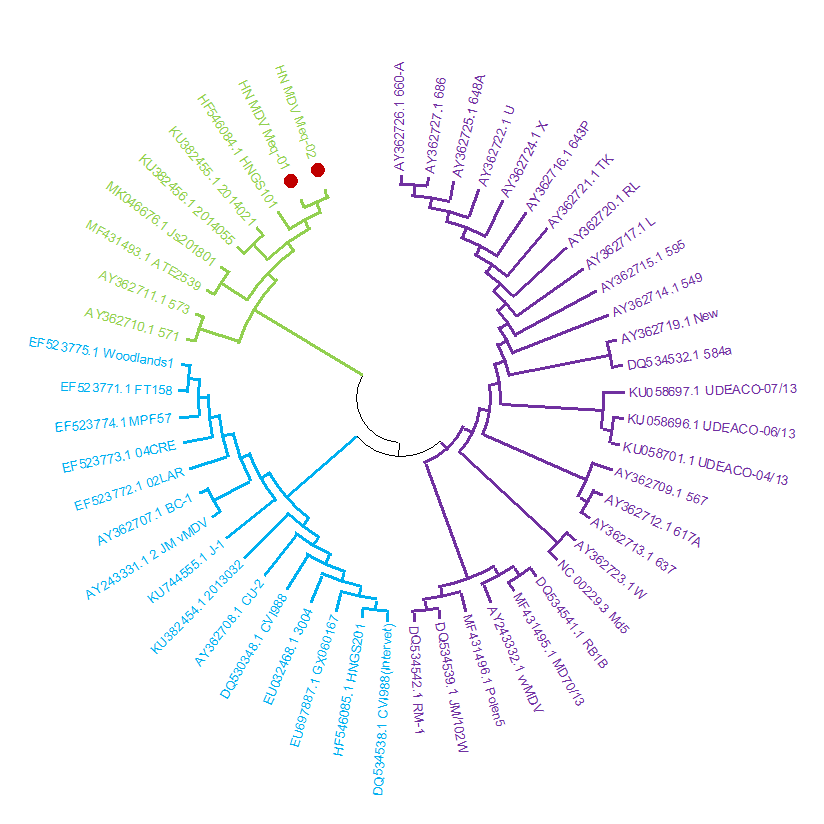


Figure 3. Phylogenetic tree assessing relationship of Hainan strains with known MDV strains. Sequences of *meq* gene that had been sequenced in this study were compared against some known strains. Dendrograms were generated by NJ method.
